# Insufficient reporting quality in large language model studies in the field of radiology

**DOI:** 10.1186/s13244-026-02236-1

**Published:** 2026-03-16

**Authors:** Pae Sun Suh, So Yeong Jeong, Daiju Ueda, Woo Hyun Shim, Hwon Heo, Chang-Yun Woo, Hyungjun Park, Chong Hyun Suh

**Affiliations:** 1 Department of Radiology, Research Institute of Radiological Science and Center for Clinical Imaging Data Science, Yonsei University College of Medicine, Seoul, Republic of Korea; 2 Department of Radiology, Seoul National University Bundang Hospital, Seoul National University College of Medicine, Seongnam, Republic of Korea; 3 Department of Artificial Intelligence, Graduate School of Medicine, Osaka Metropolitan University, Osaka, Japan; 4 Department of Radiology and Research Institute of Radiology, Asan Medical Center, University of Ulsan College of Medicine, Seoul, Republic of Korea; 5 Department of Medical Science, Asan Medical Institute of Convergence Science and Technology, Asan Medical Center, University of Ulsan College of Medicine, Seoul, Republic of Korea; 6 Department of Internal Medicine, Asan Medical Center, University of Ulsan College of Medicine, Seoul, Republic of Korea; 7 Department of Pulmonology, Shihwa Medical Center, Siheung, Republic of Korea

**Keywords:** Large language model, Radiology, Reporting quality, Systematic review

## Abstract

**Objectives:**

Our systematic review aimed to evaluate the quality of reporting in research articles involving LLMs in the radiology field.

**Materials and methods:**

After searching the PubMed-MEDLINE and EMBASE databases, a total of 246 eligible studies published between November 30, 2022, and December 31, 2024, were included. The analysis assessed the percentage of studies adhering to key elements required for LLM research, based on the MInimum reporting items for CLear Evaluation of Accuracy Reports of Large Language Models in healthcare (MI-CLEAR-LLM) and the Transparent Reporting of a Multivariable Model for Individual Prognosis Or Diagnosis-large language models (TRIPOD-LLM) checklists. Studies published before and after July 25, 2024, were compared using a chi-square test.

**Results:**

The most common topic was performance evaluation of LLMs using radiologic cases (44.3%, 109/246), followed by radiology reporting (37.8%, 93/246). Although all studies reported LLM’s name, only 27.6% (68/246) specified the model version, 35.8% (88/246) mentioned access date, and 25.2% (62/246) mentioned application programming interface usage. Full prompts were provided in 41.1% (101/246) of studies. Output probability-related issues, including the number of attempts (22.8%, 56/246) and factors such as temperature (16.7%, 41/246), were under-reported. These reporting insufficiencies persisted in studies published before and after July 25, 2024.

**Conclusion:**

Most studies assessing large language models in radiology lacked sufficient reporting of key elements required for large language model research. We recommend that authors strive to adhere to these elements to ensure transparency and improve the reproducibility of future studies.

**Critical relevance statement:**

Our study highlighted the need for improved reporting quality and adherence to key elements to ensure transparent reporting and improve the reproducibility of future studies using large language models.

**Key Points:**

- Numerous studies on large language models (LLMs) in radiology lack standardized methodologies, leading to high variability and inconsistent reporting.
- Our review demonstrated insufficiency in key elements for LLM research, particularly in model details and output probability.
- Better reporting and adherence to key elements are essential for enhancing transparency and reproducibility in future LLM research.

**Graphical Abstract:**

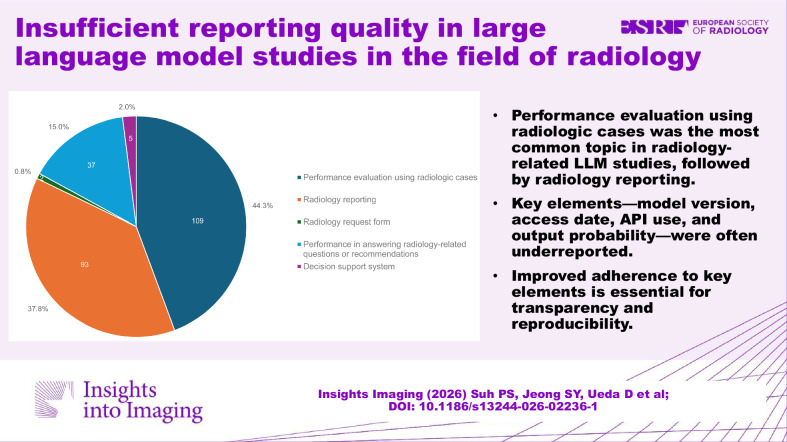

## Introduction

Large language models (LLMs) have advanced rapidly and have been applied in the field of radiology. Their text-based capabilities in natural language processing have demonstrated substantial performance in diagnostic accuracy [1, 2], radiology reporting [3–5], and providing recommendations or information [6–8]. The addition of vision capabilities has expanded their potential in the radiology field [9–11]. Numerous studies in this rapidly evolving field of medical research are being published continuously; however, their methodologies and reporting frameworks are inconsistent and highly variable due to the lack of standardized reporting guidelines [12]. These inconsistencies make it challenging for readers and reviewers to interpret study results.

Several reporting guidelines exist for artificial intelligence (AI), such as the Transparent Reporting of a multivariable prediction model of Individual Prognosis Or Diagnosis-AI (TRIPOD-AI) [13] and the Checklist for Artificial Intelligence in Medical Imaging (CLAIM) [14]. Nonetheless, these guidelines do not fully reflect the unique characteristics of LLMs compared to narrow AI. Unlike narrow AI, LLMs can generate varying outputs probabilistically, even with the exact same input, because the process involves the random sampling of a token given the output probability distribution [15]. Additionally, since LLMs are pre-trained on vast amounts of open-source data, the independence of test data—whether the data might have been included in the model’s training process—should be considered. To address this gap, new guidelines specifically focused on LLM research have recently been reported. The MInimum reporting items for CLear Evaluation of Accuracy Reports of Large Language Models in healthcare (MI-CLEAR-LLM) checklist, published online on September 12, 2024, provides essential reporting components to enhance the transparency and reporting quality of clinical studies evaluating LLMs in healthcare applications [16]. The Transparent Reporting of a Multivariable Model for Individual Prognosis Or Diagnosis-large language models (TRIPOD-LLM), published online on July 25, 2024, is an extension of TRIPOD-AI developed to address unique considerations specific to generative AI models [17]. To our knowledge, assessments of reporting quality in published articles for adherence to these guidelines are limited. Such an analysis would offer insight into the current state of reporting quality of published articles and provide valuable direction for future studies.

Our systematic review aimed to evaluate the quality of reporting in research articles involving LLMs in the field of radiology. For this purpose, we assessed using key elements based on the MI-CLEAR-LLM and TRIPOD-LLM checklists. Additionally, we sought to compare the reporting quality of research articles published before and after the introduction of these key checklists. Through these assessments, we aimed to provide an overview of the current state of reporting practices and offer recommendations to authors on how to enhance the quality of their reports.

## Materials and methods

We reported this systematic review in accordance with the Preferred Reporting Items for Systematic Reviews and Meta-Analyses (PRISMA) guidelines [18]. The protocol for this systematic review was registered with the University of York’s International Prospective Register of Systematic Reviews (PROSPERO) database (registration number CRD420250626212) and is accessible at https://www.crd.york.ac.uk/prospero/display_record.php?ID = CRD420250626212. Institutional review board approval and written informed consent were not required for our study due to its inherent nature.

### Search strategy

The PubMed-MEDLINE and EMBASE databases were searched systematically to identify published original literature reporting applications or performance of the LLMs in radiology. The following search terms were used: ([LLM] OR [“large language model*”] OR [chatgpt] OR [gpt-3.5] OR [gpt-4] OR [gpt-4o] OR [claude] OR [gemini] OR [chatbot]) AND ([radiology] OR [image] OR [imaging] OR [radiograph*] OR [X-ray] OR [“computed tomography”] OR [CT] OR [“magnetic resonance imaging”] OR [MRI] OR [ultrasound] OR [ultrasono*] OR [radiology report*] OR [CT report*] OR [MR report*]). The literature search was confined to studies published after November 30, 2022. This date was deliberately chosen to coincide with the public release of ChatGPT by OpenAI, which represented a significant turning point in the accessibility and widespread adoption of LLMs in various fields. The databases were searched for articles published on or before December 31, 2024.

### Study selection

**Fig. 1:**
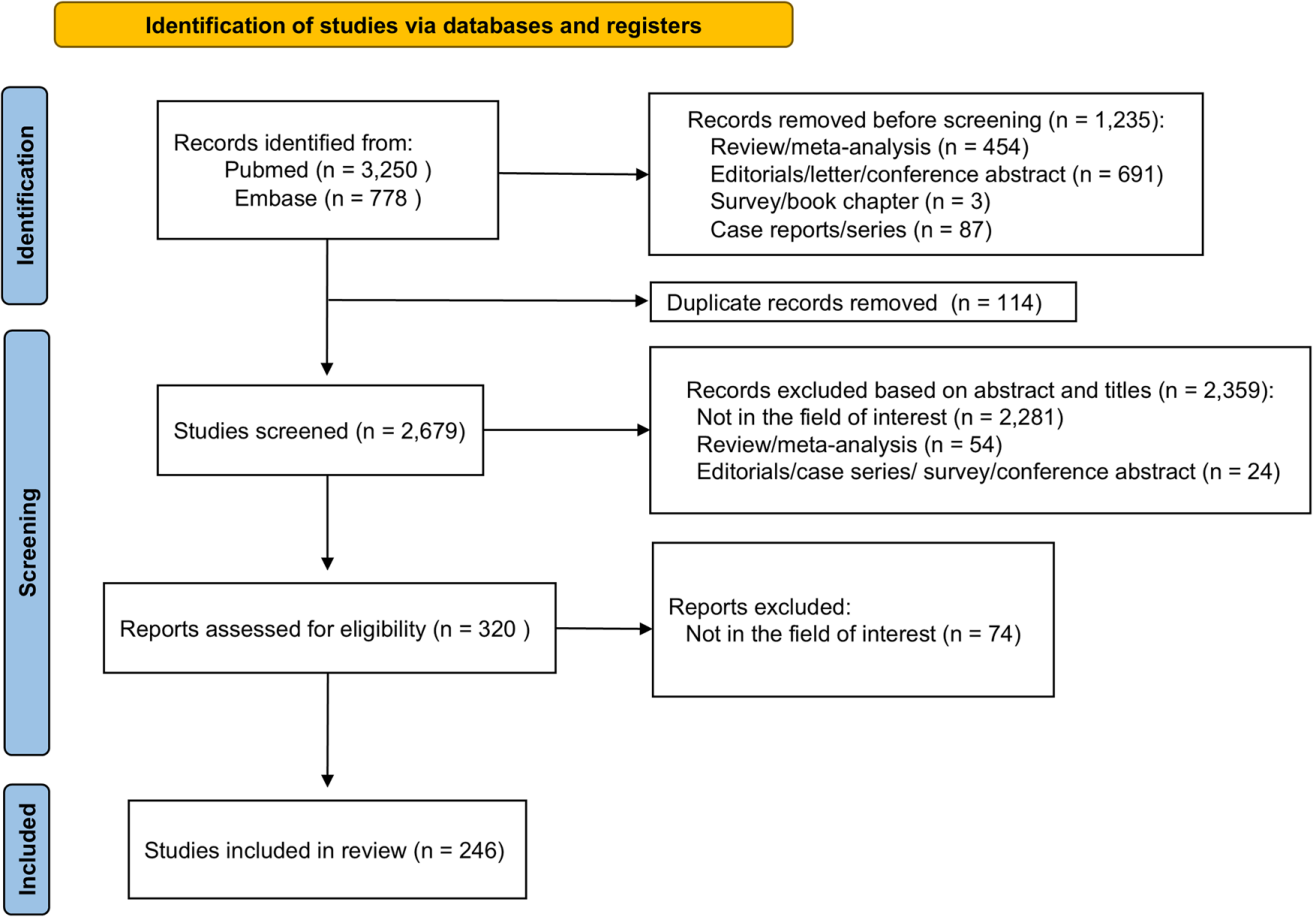
Flow diagram of the study selection process

The study selection process for the search strategy and identification of relevant studies is summarized in Fig. 1. The inclusion criteria were as follows: (1) application or performance of LLM; and (2) the field in which the LLM is applied is related to radiology. The exclusion criteria were as follows: (1) the field in which the LLM is applied is not related to radiology; and (2) conference abstracts, review articles, letters, editorials, comments, notes, short surveys, consensus statements, guidelines, or chapters. The search and study selection were performed independently by two reviewers (P.S.S. and S.Y.J., with 9 and 10 years of experience in radiology research, respectively), and any disagreements were resolved by consensus.

### Data extraction

Regarding the key elements required in LLM research, three reviewers (P.S.S., S.Y.J., and C.H.S.) discussed them based on the checklists from MI-CLEAR-LLM [16] and TRIPOD-LLM [17]. These two checklists were selected because they were the most recently available guidelines that the included studies could have feasibly referenced based on their publication dates. Subsequently, data extraction of key elements from each study was performed independently by three reviewers, and any disagreements were resolved by consensus. The extracted key elements from each study were as follows: (1) study methodology (study design, institutional review board approval), (2) test dataset (3) detailed application areas of LLM (question-answering systems, radiology reporting, radiology request forms, radiology-related questions or recommendations systems, or clinical decision support systems); (4) specifications of used LLM (model name, model version, accessing date, application programming interface (API), prompt); (5) performance metrics (accuracy, classification task performance, or test quality evaluation); (5) output probability handling (repetition, synthesis of multiple results, reliability analysis of output of repetition, temperature, top-p, top-k, frequency penalty, repetition penalty, random seed); (6) comparison (comparison with human reader, multiple comparisons methods) (7) transfer learning technique (zero shot, few shot, fine-tuning).

Publication date was defined as the earliest available date reported for each article (e.g., E-pub, ahead-of-print, or print publication date). Definitions for LLM application topics are presented in Table 1.

**Table 1:** Definitions for LLM application topics

Category	Definition
Performance evaluation using radiologic cases	Studies evaluating the diagnostic or analytical performance of LLMs using cases that include radiologic images or imaging findings
Radiology reporting	Studies in which the LLM’s primary task involved radiology reports, including generation, data extraction, summarization, or report improvement
Radiology request form	Studies in which the LLM’s primary task was to analyze, complete, or improve the appropriateness of imaging orders or request forms based on provided clinical information
Performance in answering radiology-related questions or recommendations	Studies assessing the LLM’s knowledge base by asking prompting questions related to radiology concepts, guidelines, or recommendations
Decision support system	Studies evaluating LLMs integrated into a system to provide clinical support for tasks beyond diagnosis (e.g., workflow optimization, resource management, or patient triage)

### Statistical analysis

Statistical analysis was performed using SPSS (version 22.0 for Windows; SPSS). Categorical data were summarized using frequencies and percentages. The included studies were grouped into two groups based on publication date (before July 25, 2024, and on or after July 25, 2024) and compared using Chi-square analysis. Bonferroni correction was applied to account for multiple comparisons across different key elements when comparing publications before and after checklists. July 25, 2024, was chosen as the cutoff because it marks the publication date of TRIPOD-LLM [17]. For the analysis of temporal trends, the publication date was encoded as a continuous variable by converting year and month into a single variable (e.g., July 2024 = 2024 + 7/12). Logistic regression analysis was performed using this time variable to assess linear trends over time in key binary elements (yes or no), comparing before and after July 25, 2024. A *p*-value < 0.05 was considered significant.

## Results

### Eligible studies and characteristics

The search of PubMed-MEDLINE and Embase identified 4028 articles. After adjusting for automated screening and removing duplicates, 2346 studies that did not meet the inclusion criteria were excluded based on their abstracts and titles. The full text of the remaining 320 studies was assessed, and 74 were discarded for reasons not related to the field of interest (e.g., articles about general AI models not focusing on LLMs, or application of LLMs in general medical fields rather than radiology). Finally, 246 eligible studies were included in this systematic review (Fig. 1, Supplementary Table S1).

A pre-analysis assessment of inter-rater reliability was conducted using a representative subset of 29 articles published in *Radiology* before the main data extraction and demonstrated a high level of consistency across reviewers (agreement incidence 97%; Supplementary Material).

**Fig. 2:**
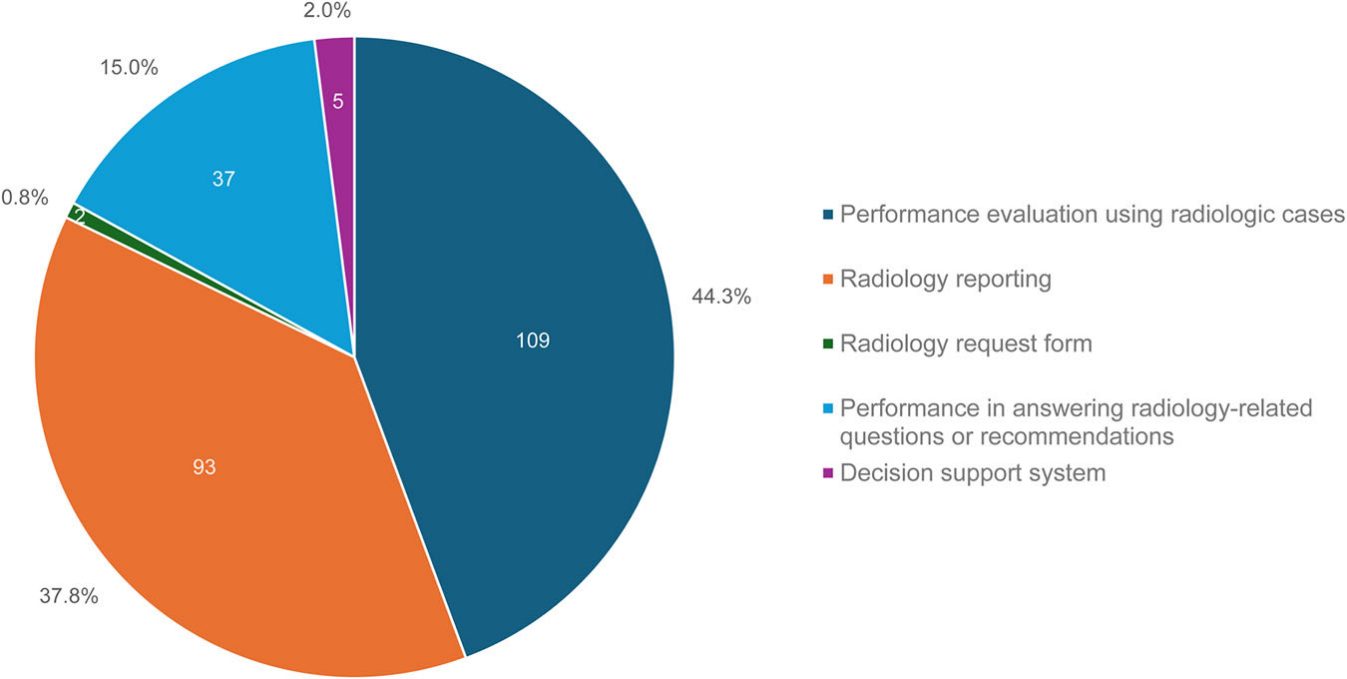
Summary of studies on large language model applications in radiology. The most common topic was the performance evaluation of LLMs using radiologic cases (44.3%, 109 of 246), followed by the use of LLMs for radiology reporting (37.8%, 93 of 246). LLM, large language model

The characteristics of the 246 included studies are presented in Fig. 2. The most common topic was the performance evaluation of LLMs using radiologic cases (44.3%, 109 of 246). The second most common topic was the use of LLMs for radiology reporting (37.8%, 93 of 246), followed by the evaluation of performance in answering radiology-related questions or providing recommendations (15.0%, 37 of 246). Only two studies reported the feasibility of using LLMs in determining radiological studies and protocols based on radiology request forms.

**Fig. 3:**
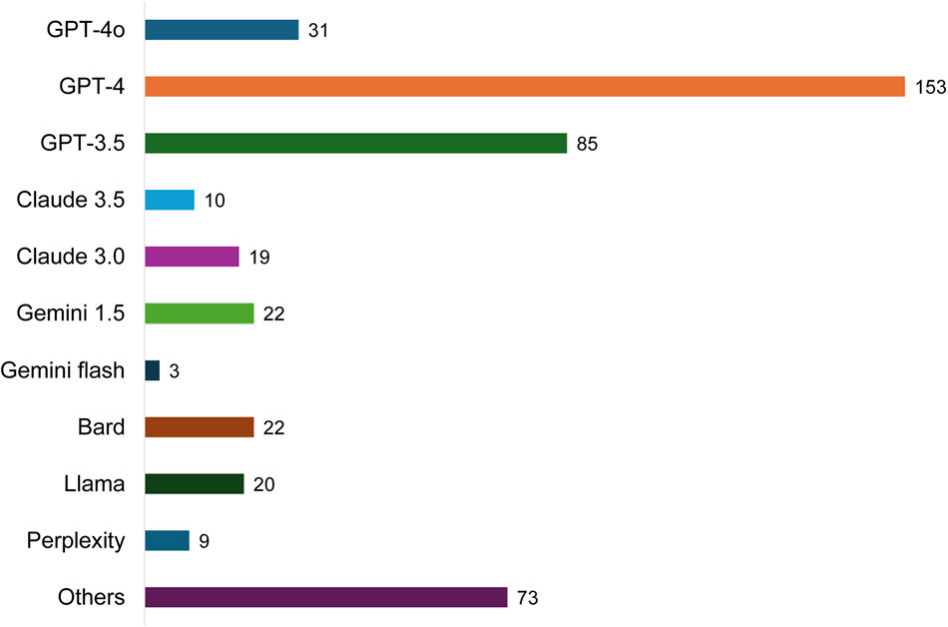
The number of large language models used in the studies. The number of LLMs was counted with consideration of overlapping data from 116 studies that assessed multiple LLMs. OpenAI’s models were the most commonly assessed models. GPT, generative pre-trained transformer; LLM, large language model

Of the 246 studies, 130 (52.8%) evaluated a single LLM, while 116 (47.2%) evaluated multiple LLMs. With consideration for overlapping data from studies that assessed more than one LLM, the models developed by OpenAI were the most commonly evaluated LLMs (GPT-4 omni: 31, GPT-4: 153, GPT-3.5: 85) (Fig. 3). Other models, such as LLaMA, were assessed in 82 studies, and two studies evaluated self-developed LLMs [19, 20].

### Adherence to key elements required for LLM research

Key elements required for LLM research based on the MI-CLEAR-LLM [16] and TRIPOD-LLM [17] checklists are described in Table 2. In terms of study design, less than half of the studies (41.5%, 102 of 246) specified whether the study was retrospective or prospective. The most common source of test data was real-world hospital data (41.5%, 102 of 246), followed by publicly available open-source data (40.2%, 99 of 246) and generated data (17.9%, 44 of 246). Prospective design studies were less likely to use real-world hospital data (Supplementary Table S2). Although all studies mentioned the name of the LLMs used, only 27.6% (68 of 246) specified the exact model version, 35.8% (88 of 246) mentioned the date of access, and 25.2% (62 of 246) mentioned the use of the API. Full prompts were provided in either the manuscripts or supplemental materials in 41.1% (101 of 246) of the studies, while 26.0% (64 of 246) did not provide prompts at all.

**Table 2:** Summary of studies on key elements required for large language model research

Section and topic	Details	No. of reported articles (percentage)			*p*-value*
		Total (*n* = 246)	Before (*n* = 124)	After (*n* = 122)	
Study methodology					
Study design	Did the study specify whether it was retrospective or prospective?	102 (41.5)	50 (40.3)	52 (42.6)	0.71
Retrospective		87 (85.3)	42 (84.0)	45 (86.5)	0.72
Prospective		15 (14.7)	8 (16.0)	7 (13.5)	0.72
IRB	Was IRB approval obtained or waived?	177 (72.0)	91 (73.4)	86 (70.5)	0.61
Obtained		92 (52.0)	39 (42.9)	53 (61.6)	0.01
Waived		85 (48.0)	52 (57.1)	33 (38.4)	0.01
Test dataset					
Public	Publicly available open-source data	99 (40.2)	49 (39.5)	50 (41.0)	0.81
Hospital	Real-world hospital data	102 (41.5)	47 (37.9)	55 (45.1)	0.25
Generated	Generated data	44 (17.9)	29 (23.4)	15 (12.3)	0.02
Others	Using publicly unavailable data or generating outputs without a test dataset	10 (4.1)	5 (4.0)	5 (4.1)	0.97
Large language models					
Model name	Did the study specify the name of the model used?	246 (100)	124 (100)	122 (100)	> 0.99
Model version	Did the study specify the exact version of the model used?	68 (27.6)	33 (26.6)	35 (28.7)	0.71
Accessing date	Did the study mention the date when the LLM was run?	88 (35.8)	46 (37.1)	42 (34.4)	0.66
API	Did the study mention whether an API was used?	62 (25.2)	33 (26.6)	29 (23.8)	0.61
Prompts					
Full prompts	Did the study provide the full prompts used in the research?	101 (41.1)	64 (51.6)	37 (30.3)	**< 0.001**
Partially provided	Providing examples of prompts without including the full prompts.	81 (32.9)	32 (25.8)	49 (40.2)	0.02
Not provided		64 (26.0)	28 (22.6)	36 (29.5)	0.22
Output probability					
Repetition	Did the study specify the number of querying attempts?	56 (22.8)	34 (27.4)	22 (18.0)	0.08
Repeated		51 (91.1)	30 (88.2)	21 (95.5)	0.35
Multiple results analysis	If repeated, did the study explain how the multiple results were synthesized for analysis?	42 (82.4)	26 (86.7)	16 (76.2)	0.34
Reliability analysis	If repeated, did the study analyze the reliability between responses?	28 (54.9)	15 (50.0)	13 (61.9)	0.41
Temperature, Top-p, Top-k, Frequency penalty, Repetition penalty, Random seed	Was there mention of the used factors related to outcome probability?	41 (16.7)	17 (13.7)	24 (19.4)	0.31
Comparison					
Human readers	Did the study compare the results with human readers?	47 (19.1)	18 (14.5)	29 (23.8)	0.06
Blinding of readers	If human readers were involved, did the study mention whether they were blinded to the data and how blinding was done?	20 (42.6)	9 (50.0)	11 (37.9)	0.42
Multiple comparison	Were three or more readers (LLM + human) involved in the comparison?	82 (33.3)	38 (30.6)	44 (36.1)	0.36
Statistical method	If multiple readers were involved, did the study consider a statistical method for multiple comparison?	11 (13.4)	7 (18.4)	4 (9.1)	0.22
*p*-value correction	If multiple readers were involved, did the study adjusted *p*-value?	15 (18.3)	10 (26.3)	5 (11.4)	0.08

*API* application programming interface, *IRB* institutional review board, *LLM* large language model

* *p*-value for comparison between studies published before and after publication of the checklist using chi-square test. A *p*-value less than 0.002 (0.05/27) was considered statistically significant after Bonferroni correction (bold values)

Regarding output probability, only 22.8% (56 of 246) mentioned the number of querying attempts, and 13.8% (34 of 246) addressed temperature, top-p, top-k, frequency penalty, repetition penalty and random seed, a parameter closely related to output probability. Among the 51 studies that performed repeated querying attempts, 54.9% (28 of 51) analyzed the reliability between the generated responses [8, 21–34]. Only 19.1% (47 of 246) of the studies compared the performance of LLMs with that of humans.

Furthermore, statistical methods used for comparison were also found to be insufficient. When comparing three or more readers, only 18.3% (15 of 82) considered *p*-value correction [9, 11, 35–47] and 13.4% (11 of 82) used specific statistical methods to account for multiple comparisons [8, 11, 22, 37, 38, 40, 41, 45–48]. Statistical methods included Cochran’s Q test in six studies, generalized mixed effects models in three, and generalized estimating equations in two studies.

Adherence to key elements required for LLM research was compared between studies published before and after the publication of the TRIPOD-LLM checklist (July 25, 2024). Approval of Institutional Review Board (IRB) was significantly more frequent in studies after checklist publication (before vs. after, 42.9% [39 of 124] vs. 61.6% [53 of 122], *p* = 0.01). Studies that provide full prompts were statistically significantly less frequent in studies after checklist publication (51.6% [64 of 124] vs. 30.3% [37 of 122]; *p* < 0.001). No other key elements showed statistically significant differences after correction for multiple comparison between studies published before July 25, 2024, and after July 25, 2024.

**Fig. 4:**
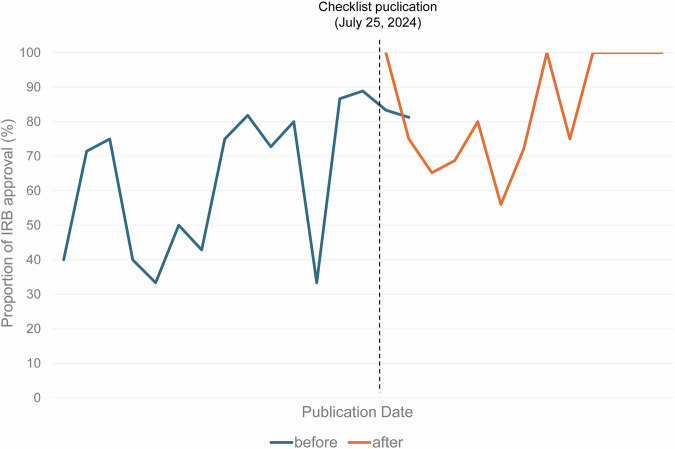
Temporal change in the proportion of studies reporting IRB approval before and after checklist publication. Before checklist publication, IRB approval shows a significant increasing trend over time (β = 1.562, *p* = 0.005). After checklist publication, the proportion of studies reporting IRB approval remained high without a further significant trend (β = 0.300, *p* = 0.764)

Temporal trends were also evaluated for studies published before and after the publication of the TRIPOD-LLM checklist (Table 3). Only the approval of IRB showed a significant increasing trend over time before checklist publication. After checklist publication, the proportion of studies reporting IRB approval remained high without a further significant trend (Fig. 4). No other key elements showed statistically significant temporal trends in either period.

**Table 3:** Temporal trend analysis in reporting key elements

Section and topic	Details	Before		After	
		β	*p*-value	β	*p*-value
Study methodology					
Study design	Did the study specify whether it was retrospective or prospective?	0.112	0.818	−0.242	0.789
IRB	Was IRB approval obtained or waived?	1.562	0.005	0.300	0.764
Large language models					
Model name	Did the study specify the name of the model used?	NA	NA	NA	NA
Model version	Did the study specify the exact version of the model used?	−0.043	0.936	0.766	0.420
Accessing date	Did the study mention the date when the LLM was run?	0.066	0.895	0.333	0.721
API	Did the study mention whether an API was used?	1.188	0.051	−0.116	0.913
Full prompts	Did the study provide the full prompts used in the research?	0.273	0.570	−0.254	0.797
Output probability					
Repetition	Did the study specify the number of querying attempts?	0.190	0.726	0.342	0.759
Temperature, Top-p, Top-k, Frequency penalty, Repetition penalty, Random seed	Was there mention of the used factors related to outcome probability?	1.555	0.068	0.539	0.657
Comparison					
Human readers	Did the study compare the results with human readers?	0.118	0.864	1.491	0.134
Multiple comparison	Were three or more readers (LLM + human) involved in the comparison?	0.761	0.165	0.726	0.427

Logistic regression analysis was performed to assess linear trends over time in key binary elements. The publication date was encoded as a continuous variable by converting year and month into a single variable (e.g., July 2024 = 2024 + 7/12)

*API* application programming interface, *IRB* institutional review board, *LLM* large language model

### Studies assessing various aspects of radiology reporting

A total of 93 studies assessed LLMs’ ability in various aspects of radiology reporting (Table 4). After categorizing them into seven topics, these 93 studies covered 99 topics, as 6 studies assessed multiple topics. Data extraction was the most frequently assessed topic, accounting for 30.3% (30 of 99), followed by conclusion/impression generation and readability enhancement (17.2%, 17 of 99), and categorization (15.2%, 15 of 99). There was no statistically significant difference in topics between the articles published before and after the publication of the checklist. However, articles about data extraction were increasingly published after checklist publication (before vs. after, 22.6% [12 of 53] vs. 39.1% [18 of 46]; *p* = 0.08) [49–66].

**Table 4:** Summary of studies assessing various aspects of radiology reporting

Topic	No. of reported articles (percentage)	No. of assessed topics (percentage)*			*p*-value^†^
	Total (*n* = 93)	Total (*n* = 99)	Before (*n* = 53)	After (*n* = 46)	
Structured reports generation	9 (9.7)	9 (9.1)	5 (9.4)	4 (8.7)	0.90
Conclusion/impression generation	13 (14.0)	17 (17.2)	10 (18.9)	7 (15.2)	0.63
Data extraction	28 (30.1)	30 (30.3)	12 (22.6)	18 (39.1)	0.08
Error detection	6 (6.5)	6 (6.1)	3 (5.7)	3 (6.5)	0.87
Categorization	13 (14.0)	15 (15.2)	11 (20.8)	4 (8.7)	0.10
Readability enhancement	14 (15.1)	17 (17.2)	9 (17.0)	8 (17.4)	0.96
Draft report generation	4 (4.3)	5 (5.1)	3 (5.7)	2 (4.3)	0.75
Multiple reporting topics					
Conclusion/impression generation and readability enhancement	3 (3.2)				
Data extraction and categorization	2 (2.2)				
Conclusion/impression generation and draft report generation	1 (1.1)				

* The number of topics assessed in 89 studies includes overlapping data from studies that evaluated multiple reporting topics

^†^ *p*-value for comparison between studies published before and after publication of the checklist using chi-square test

Among the 93 studies, only 21.5% (20 of 93) specified the exact model version and the date of access. Full prompts were provided in 43.0% (40 of 93) of the studies. Mention of used factors related to output probability, including temperature, top-p, top-k, frequency penalty, repetition penalty and random seed, was only included in 17.2% (16 of 93) of the studies.

### Studies evaluating LLM performance using radiologic cases

A total of 109 studies evaluating LLM performance using radiologic cases were included in this systematic analysis (Table 5). The number of cases used as inputs ranged from 5 to 32,951 (median [interquartile range], 134 [100–230]). After LLMs with vision capability were released in late 2023, image inputs were evaluated in 50.5% (55 of 109) of the studies, without a significant change in studies before and after checklist publication (before vs. after, 51.0% [25 of 49] vs. 50.0% [30 of 60]; *p* = 0.92). Majority of studies (65.1%, 71 of 109) used public datasets without any rephrasing. Most studies (80.7%, 88 of 109) assessed LLM performance by reporting diagnostic accuracy. Additionally, 14 studies reported sensitivity and specificity, and eight studies assessed the quality of output using a five-point Likert scale.

**Table 5:** Summary of studies evaluating large language model performance using radiologic cases

Section and topic	No. of reported articles (percentage)			*p*-value^†^
	Total (*n* = 109)	Before (*n* = 49)	After (*n* = 60)	
Input				
With image inputs	55 (50.5)	25 (51.0)	30 (50.0)	0.92
Without image inputs	54 (49.5)	24 (49.0)	30 (50.0)	0.92
Number of cases*	134 (100–230)	139 (102–205)	130 (85–238)	0.10
Test dataset				
Public	71 (65.1)	34 (69.4)	37 (61.7)	0.40
Rephrasing dataset	0 (0)	0 (0)	0 (0)	> 0.99
Hospital	29 (26.6)	9 (18.4)	20 (33.3)	0.08
Generated	12 (11.0)	8 (16.3)	4 (6.7)	0.11
Others	1 (0.9)	1 (2.0)	0 (0)	0.27
Evaluation				
Accuracy	88 (80.7)	36 (73.5)	52 (86.7)	0.08
Sensitivity, specificity	14 (12.8)	8 (16.3)	6 (10.0)	0.33
Five-point Likert scale	8 (7.3)	5 (10.2)	3 (5.0)	0.30
Comparison with human readers	29 (26.6)	10 (20.4)	19 (31.7)	0.19
Number of human readers*	3 (2–6)	3 (2–6)	3 (3–6)	0.15
Blinding of human readers	11 (37.9)	5 (50.0)	6 (31.6)	0.34
Multiple readers (≥ 3)	49 (45.0)	19 (38.8)	30 (50.0)	0.24
Statistical methods for multiple comparison	9 (18.4)	5 (26.3)	4 (13.3)	0.26
Cochran’s Q test	6 (66.7)	3 (60.0)	3 (75.0)	0.65
Generalized estimating equations	3 (33.3)	2 (40.0)	1 (25.0)	0.65
*p*-value correction	11 (22.4)	6 (31.6)	5 (16.7)	0.23

* Data are presented as median (interquartile range)

^†^ Comparison of the number of cases between studies published before and after publication of the checklist using an independent t-test. Otherwise, comparison was performed using chi-square test

Three or more readers, including both LLMs and humans, were involved in 49 studies. Among these, only 18.4% (9 of 49) used statistical methods to account for multiple comparisons (Cochran’s Q test in six studies [22, 41, 45–48], generalized estimating equations in two [11, 37], and generalized mixed effects models in one [40]), and 22.4% (11 of 109) adjusted the *p*-value to account for multiple comparisons [9, 11, 37, 39–42, 44–47]. Human readers were involved and compared with LLMs in 26.6% (29 of 109) of the studies, with the number of human readers ranging from 1 to 63 (median [interquartile range], 3 [2–6]). Only 37.9% (11 of 29) of these studies mentioned whether the human readers were blinded to the data and how blinding was performed.

### Studies evaluating radiology-related questions or recommendation systems

A total of 36 studies evaluated LLMs’ performance on radiology-related questions or recommendation systems. The number of questions used as inputs ranged from 3 to 1633. LLMs’ performance was assessed by reporting text quality in 58.3% (21 of 36), diagnostic accuracy in 36.1% (13 of 36), or classification task performance in 5.6% (2 of 36). Regarding output probability, only 27.8% (10 of 36) performed repeated querying attempts, and only two studies addressed temperature [67, 68]. The datasets used were diverse: public data in 30.6% (11 of 36), hospital data in 19.4% (7 of 36), newly generated data in 41.7% (15 of 36), and others in 11.1% (4 of 36). The most commonly used LLMs were OpenAI’s models (28 of 36), and 44.4% (16 of 36) of the studies used two or more LLMs in their research.

## Discussion

Our systematic review focused on the reporting quality of the rapidly growing number of published articles on large language models in the field of radiology. Numerous studies have assessed the abilities of large language models in various topics, such as radiology reporting, question-answering on radiologic cases, and providing recommendations. However, regarding key elements based on the MI-CLEAR-LLM and TRIPOD-LLM checklists, several limitations were identified, particularly in dealing with detailed information about large language models used, output probability, and statistical methods for multiple comparisons. These limitations appeared to persist without immediate change in studies published after July 25, 2024, compared to earlier publications. Our study highlighted the importance of adhering to these key elements to ensure transparent reporting and improve the reproducibility of future studies using large language models.

Transparency in LLM settings related to output probability is crucial to prevent the risk of selectively reporting favorable results. Essential elements for managing output probability include the number of querying attempts, methods for synthesizing multiple responses, and reliability analysis across attempts [16]. A recent systematic review revealed that only 15.1% of high-quality studies assessing LLMs for medical applications clearly reported output probability-related issues [69]. Similarly, our study found that only 22.8% of studies assessing LLMs in radiology reported the number of querying attempts, and just half of these studies conducted further reliability analysis. Temperature is a parameter associated with the model’s output probabilities; a higher temperature increases randomness and creativity, while a lower temperature increases the determinism of the outputs [70]. It was also overlooked, with only 13.8% of studies mentioning the temperature setting. Consequently, as temperature is closely related to output probability and is an adjustable parameter, the use of APIs is increasingly important. Given that temperature was by far the most frequently reported and discussed parameter, other generation parameters such as top-p, top-k, repetition penalties, and random seed were not evaluated in this study. The reporting of these parameters was also limited, which made a comprehensive analysis infeasible. We recommend that future studies provide full transparency by reporting all generation parameters. In addition, future studies could explore adjusting model parameters to make LLMs more deterministic, which would be crucial for ensuring safe and consistent clinical applications.

Moreover, different LLMs and versions can generate different responses to the same query, owing to differences in parameters, training data, or fine-tuning methods. As LLMs are updated rapidly and continuously, replicating study results becomes extremely difficult [71]. Therefore, it is essential for studies to precisely specify the name, version, and access date of the LLMs used. Although all the studies in our review mentioned the LLMs’ names, only 27.6% and 35.8% reported the specific model version and access date, respectively. Additionally, prompting is another critical factor because even minor changes to prompts can result in different outputs [72]. Although about half of the studies provided full prompts, it is surprising that 26.0% of studies did not provide any prompts at all. Our study revealed substantial deficiencies in reporting factors affecting outputs, including output probability, prompting, and details of LLMs used. Addressing these gaps highlights the importance of specifying these factors to improve transparency and reproducibility in LLM studies.

Our study categorized the topics assessed in the studies, and the most common topic was the evaluation of LLMs’ performance using radiologic cases. With the development of multimodal LLMs, evaluation using radiologic image inputs has been significantly increasing. However, when evaluating using cases from open-source data, caution is needed for data leakage [71, 73]. The independence of test data should be considered in interpreting the study results. Most of the studies (65.1%) used open-source public data, but only 26.6% of studies used real-world hospital data; notably, the use of real-world hospital data increased substantially from 18.4% before July 25, 2024, to 33.3% after this date. When using open-source data, it is essential to consider independence, and ultimately, the focus of LLM study should shift toward evaluating real-world data in the future. The second most common topic was radiology reporting. Various aspects of radiology reporting were assessed in 93 studies, with a recent increase in the number of publications. Clinical application of LLMs in radiology reporting is promising in improving the quality of reporting, reducing effort and time for radiologists, and leading to reduced burnout [70, 74].

Including multiple LLMs or human readers in performance evaluation and comparison has recently increased significantly, raising the need for specific statistical considerations, especially when assessing performance on quiz cases. In our study, 33.3% of studies involved three or more readers; however, only 18.3% applied *p*-value corrections, and 13.4% used specific statistical methods, such as Cochran’s Q test or generalized mixed effects models. Cochran’s Q test is effective for comparing proportions across three or more groups [75]. Generalized mixed effects models [76] and generalized estimating equations [77] are statistical methods that can account for the dependence inherent in measurements across multiple timepoints. Additionally, post-hoc pairwise tests with adjustments like Bonferroni are essential to account for multiple comparisons [78]. Notably, four studies stated that multiplicity adjustments were omitted because they were exploratory studies. Since the most appropriate statistical method varies with study design, authors must consult expert statisticians to enhance the study quality.

There are several limitations in this study. First, our study evaluated the reporting quality of studies based on key elements from the MI-CLEAR-LLM [16] and TRIPOD-LLM [17] checklists. However, guidelines for LLMs in healthcare applications are still evolving and not yet fully established. In addition, our study focused on the reporting quality related to LLM use, and this does not indicate the reporting quality of each study itself. Second, given that the objective of our review was centered on the clinical focus of LLMs in the radiology field, we confined our searching to PubMed/MEDLINE and Embase and did not include broader sources such as IEEE and arXiv to ensure the inclusion of clinically validated literature. Third, our study analyzed multiple subcategories and compared studies published before July 25, 2024, and on or after July 25, 2024. The small proportion of the studies adhering to key elements within each subcategory limits the number of articles available for comparison, which may reduce statistical power. Additionally, there is an inherent temporal limitation in our study design. By using the publication date rather than the submission date as the cutoff, our analysis may underestimate the true adoption lag of the guidelines, as manuscripts published shortly after the guideline release were unlikely to have incorporated the new recommendations. Additionally, given the short time interval between guideline publication and the end of the inclusion period, definitively measuring the immediate impact of guideline adoption is difficult. Nevertheless, the continuous observation of reporting insufficiencies in studies published as late as December 2024 suggests that the overall findings of our systematic review remain highly relevant. Fourth, given the exponential growth in LLM research publications, our systematic review may have limitations in comprehensively capturing all developments in this rapidly evolving field up to the present time. Although we made extensive efforts to include the most recent studies, the dynamic and fast-paced nature of LLM research presents inherent challenges in providing a completely up-to-date synthesis of the literature. Additionally, as research is ongoing in 2025, the studies we analyzed from this year may not fully represent the reporting quality of all 2025 publications.

In conclusion, the majority of studies assessing large language models in radiology lacked sufficient reporting of key elements required for large language model research, particularly in areas of output probability, detailed model specifications, and specific statistical methods. We recommend that authors strive to adhere to these elements to ensure transparent reporting and improve the reproducibility of future studies using large language models.

## Supplementary information

ELECTRONIC SUPPLEMENTARY MATERIAL

## Data Availability

Data generated or analyzed during the study are available from the corresponding author upon request.

## Abbreviations

API: Application programming interface
AI: Artificial intelligence
GPT: Generative pre-trained transformer
LLM: Large language model
MI-CLEAR-LLM: MInimum reporting items for CLear Evaluation of Accuracy Reports of Large Language Models in healthcare
TRIPOD-LLM: Transparent Reporting of a Multivariable Model for Individual Prognosis Or Diagnosis-large language models
