# Supplementary material for: Insufficient reporting quality in large language model studies in the field of radiology
Source: Insights Imaging. 2026 Mar 16;17:71. doi: 10.1186/s13244-026-02236-1 (PMC12992711; doi:10.1186/s13244-026-02236-1)
Supplement: Supplementary file 1 — ELECTRONIC SUPPLEMENTARY MATERIAL [file 13244_2026_2236_MOESM1_ESM.pdf]

Insufficient Reporting Quality in Large Language Model Studies in the Field of Radiology

ELECTRONIC SUPPLEMENTARY MATERIAL

Supplementary Table 1. List of 246 studies analyzed

No.	First Author	Year	Title	Journal
1	Ariyaratne S et al	2024	Could ChatGPT Pass the UK Radiology Fellowship Examinations?	Acad Radiol
2	Kooraki S et al	2024	Evaluation of ChatGPT-Generated Educational Patient Pamphlets for Common Interventional Radiology Procedures	Acad Radiol
3	Mistry NP et al	2024	Large Language Models as Tools to Generate Radiology Board-Style Multiple-Choice Questions	Acad Radiol
4	Nguyen D et al	2024	Evaluation of ChatGPT and Google Bard Using Prompt Engineering in Cancer Screening Algorithms	Acad Radiol
5	Payne DL et al	2024	Performance of GPT-4 on the American College of Radiology In-training Examination: Evaluating Accuracy, Model Drift, and Fine-tuning	Acad Radiol
6	Al-Naser Y et al	2025	Evaluating Artificial Intelligence Competency in Education: Performance of ChatGPT-4 in the American Registry of Radiologic Technologists (ARRT) Radiography Certification Exam	Acad Radiol
7	Beşler MS et al	2024	Evaluating GPT-4o's Performance in the Official European Board of Radiology Exam: A Comprehensive Assessment	Acad Radiol
8	Can E et al	2025	Large Language Models for Simplified Interventional Radiology Reports: A Comparative Analysis	Acad Radiol
9	Ghosh A et al	2025	Large Language Models can Help with Biostatistics and Coding Needed in Radiology Research	Acad Radiol
10	Han NY et al	2025	Enhancing Oncological Surveillance Through Large Language Model-Assisted Analysis: A Comparative Study of GPT-4 and Gemini in Evaluating Oncological Issues From Serial Abdominal CT Scan Reports	Acad Radiol
11	Kaba E et al	2025	Accuracy and Readability of ChatGPT on Potential Complications of Interventional Radiology Procedures: AI-Powered Patient Interviewing	Acad Radiol
12	Nakaura T et al	2025	Performance of Multimodal Large Language Models in Japanese Diagnostic Radiology Board Examinations (2021-2023)	Acad Radiol
13	Soleimani M et al	2024	Practical Evaluation of ChatGPT Performance	Acad Radiol

			for Radiology Report Generation	
14	Sun SH et al	2025	Large Language Models with Vision on Diagnostic Radiology Board Exam Style Questions	Acad Radiol
15	Xu J et al	2024	Performance of ChatGPT and Radiology Residents on Ultrasonography Board-Style Questions	Advanced Ultrasound in Diagnosis and Therapy
16	Chien A et al	2024	AI-Assisted Summarization of Radiologic Reports: Evaluating GPT3davinci, BARTcnn, LongT5booksum, LEDbooksum, LEDlegal, and LEDclinical	AJNR Am J Neuroradiol
17	Kikuchi T et al	2024	Towards Improved Radiological Diagnostics: Investigating the Utility and Limitations of GPT-3.5 Turbo and GPT-4 with Quiz Cases	AJNR Am J Neuroradiol
18	Kikuchi T et al	2024	Toward Improved Radiologic Diagnostics: Investigating the Utility and Limitations of GPT-3.5 Turbo and GPT-4 with Quiz Cases	AJNR Am J Neuroradiol
19	Bhayana R et al	2024	Use of GPT-4 With Single-Shot Learning to Identify Incidental Findings in Radiology Reports	AJR Am J Roentgenol
20	Cao JJ et al	2023	Accuracy of Information Provided by ChatGPT Regarding Liver Cancer Surveillance and Diagnosis	AJR Am J Roentgenol
21	Haver HL et al	2024	Use of ChatGPT to Assign BI-RADS Assessment Categories to Breast Imaging Reports	AJR Am J Roentgenol
22	Lee JE et al	2024	Lung Cancer Staging Using Chest CT and FDG PET/CT Free-Text Reports: Comparison Among Three ChatGPT Large Language Models and Six Human Readers of Varying Experience	AJR Am J Roentgenol
23	Young CC et al	2025	Diagnostic Accuracy of a Custom Large Language Model on Rare Pediatric Disease Case Reports	Am J Med Genet A
24	Sievert M et al	2024	Risk stratification of thyroid nodules: Assessing the suitability of ChatGPT for text-based analysis	Am J Otolaryngol
25	Eghbali N et al	2024	Improving Automating Quality Control in Radiology: Leveraging Large Language Models to Extract Correlative Findings in Radiology and Operative Reports	AMIA Jt Summits Transl Sci Proc
26	Kartsonis W et al	2025	Enhancing Aortic Aneurysm Surveillance: Transformer Natural Language Processing for Flagging and Measuring in Radiology Reports	Ann Vasc Surg
27	Cesur T et al	2024	Use of large language models in radiological reports: A study on simplifying turkish MRI findings	Annals of Clinical and Analytical Medicine
28	Mert S et al	2024	Diagnostic power of ChatGPT 4 in distal radius fracture detection through wrist radiographs	Arch Orthop Trauma Surg
29	Bergomi L et al	2024	Reshaping free-text radiology notes into structured reports with generative question answering transformers	Artif Intell Med
30	Liu J et al	2025	Generating synthetic clinical text with local large language models to identify misdiagnosed limb	Artif Intell Med

			fractures in radiology reports	
31	Jin Q et al	2024	Hidden Flaws Behind Expert-Level Accuracy of Multimodal GPT-4 Vision in Medicine	ArXiv
32	Lanfredi RB et al	2024	Enhancing chest X-ray datasets with privacy-preserving large language models and multi-type annotations: a data-driven approach for improved classification	ArXiv
33	Voinea Ș V et al	2024	GPT-Driven Radiology Report Generation with Fine-Tuned Llama 3	Bioengineering (Basel)
34	Serapio A et al	2024	An open-source fine-tuned large language model for radiological impression generation: a multi-reader performance study	BMC Med Imaging
35	Dong R et al	2024	Classification of lumbar spine disorders using large language models and MRI segmentation	BMC Med Inform Decis Mak
36	Gamble JL et al	2024	Limitations of GPT-3.5 and GPT-4 in Applying Fleischner Society Guidelines to Incidental Lung Nodules	Can Assoc Radiol J
37	Patil NS et al	2024	Comparative Performance of ChatGPT and Bard in a Text-Based Radiology Knowledge Assessment	Can Assoc Radiol J
38	Wagner MW et al	2024	Accuracy of Information and References Using ChatGPT-3 for Retrieval of Clinical Radiological Information	Can Assoc Radiol J
39	Patil NS et al	2024	Artificial Intelligence Chatbots' Understanding of the Risks and Benefits of Computed Tomography and Magnetic Resonance Imaging Scenarios	Can Assoc Radiol J
40	Sun D et al	2024	Outcome Prediction Using Multi-Modal Information: Integrating Large Language Model-Extracted Clinical Information and Image Analysis	Cancers (Basel)
41	Matsuo H et al	2024	Exploring Multilingual Large Language Models for Enhanced TNM Classification of Radiology Report in Lung Cancer Staging	Cancers (Basel)
42	Scheschenja M et al	2024	Feasibility of GPT-3 and GPT-4 for in-Depth Patient Education Prior to Interventional Radiological Procedures: A Comparative Analysis	Cardiovasc Intervent Radiol
43	Zaki HA et al	2024	Using ChatGPT to Improve Readability of Interventional Radiology Procedure Descriptions	Cardiovasc Intervent Radiol
44	Monroe CL et al	2024	Evaluation of responses to cardiac imaging questions by the artificial intelligence large language model ChatGPT	Clin Imaging
45	Nguyen D et al	2024	Encouragement vs. liability: How prompt engineering influences ChatGPT-4's radiology exam performance	Clin Imaging
46	Nguyen D et al	2025	Exploring the accuracy of embedded ChatGPT-4 and ChatGPT-4o in generating BI-RADS scores: a pilot study in radiologic clinical support	Clin Imaging
47	Kozel G et al	2024	Chat-GPT on brain tumors: An examination of Artificial Intelligence/Machine Learning's ability to provide diagnoses and treatment plans for	Clin Neurol Neurosurg

			example neuro-oncology cases	
48	Horiuchi D et al	2024	Comparing the Diagnostic Performance of GPT-4-based ChatGPT, GPT-4V-based ChatGPT, and Radiologists in Challenging Neuroradiology Cases	Clin Neuroradiol
49	Silva TP et al	2024	Performance of a commercially available Generative Pre-trained Transformer (GPT) in describing radiolucent lesions in panoramic radiographs and establishing differential diagnoses	Clin Oral Investig
50	Gupta M et al	2024	Can generative AI improve the readability of patient education materials at a radiology practice?	Clin Radiol
51	Cesur T et al	2024	Optimizing Diagnostic Performance of ChatGPT: The Impact of Prompt Engineering on Thoracic Radiology Cases	Cureus
52	Mago J et al	2023	The Potential Usefulness of ChatGPT in Oral and Maxillofacial Radiology	Cureus
53	Suthar PP et al	2023	Artificial Intelligence (AI) in Radiology: A Deep Dive Into ChatGPT 4.0's Accuracy with the American Journal of Neuroradiology's (AJNR) "Case of the Month"	Cureus
54	Tepe M et al	2024	Assessing the Responses of Large Language Models (ChatGPT-4, Gemini, and Microsoft Copilot) to Frequently Asked Questions in Breast Imaging: A Study on Readability and Accuracy	Cureus
55	Abdul Sami M et al	2024	ChatGPT-4 Turbo and Meta's LLaMA 3.1: A Relative Analysis of Answering Radiology Text-Based Questions	Cureus
56	Abdul Sami M et al	2024	Comparative Accuracy of ChatGPT 4.0 and Google Gemini in Answering Pediatric Radiology Text-Based Questions	Cureus
57	Asari Y et al	2024	This Is a Quiz Premise Input: A Key to Unlocking Higher Diagnostic Accuracy in Large Language Models	Cureus
58	Goto H et al	2024	Performance Evaluation of GPT-4o and o1-Preview Using the Certification Examination for the Japanese 'Operations Chief of Radiography With X-rays'	Cureus
59	Gupta R et al	2024	Comparative Evaluation of AI Models Such as ChatGPT 3.5, ChatGPT 4.0, and Google Gemini in Neuroradiology Diagnostics	Cureus
60	Ishida M et al	2024	Diagnostic Performance of GPT-4o and Claude 3 Opus in Determining Causes of Death From Medical Histories and Postmortem CT Findings	Cureus
61	Javan R et al	2024	Programming Chatbots Using Natural Language: Generating Cervical Spine MRI Impressions	Cureus
62	Kanzawa J et al	2024	Evaluating the Role of GPT-4 and GPT-4o in the Detectability of Chest Radiography Reports Requiring Further Assessment	Cureus
63	Karnan N et al	2024	Analyzing the Effectiveness of AI-Generated	Cureus

			Patient Education Materials: A Comparative Study of ChatGPT and Google Gemini	
64	Lotfian G et al	2024	Evaluation of ChatGPT 4.0 in Thoracic Imaging and Diagnostics	Cureus
65	Bosbach WA et al	2024	Ability of ChatGPT to generate competent radiology reports for distal radius fracture by use of RSNA template items and integrated AO classifier	Curr Probl Diagn Radiol
66	Doddi S et al	2024	Assessing appropriate responses to ACR urologic imaging scenarios using ChatGPT and Bard	Curr Probl Diagn Radiol
67	Scheschenja M et al	2024	ChatGPT: Evaluating answers on contrast media related questions and finetuning by providing the model with the ESUR guideline on contrast agents	Curr Probl Diagn Radiol
68	Bala W et al	2025	Enhancing radiology training with GPT-4: Pilot analysis of automated feedback in trainee preliminary reports	Curr Probl Diagn Radiol
69	Hofmann HL, Vairavamurthy J	2024	Large language model doctor: assessing the ability of ChatGPT-4 to deliver interventional radiology procedural information to patients during the consent process	CVIR Endovasc
70	Elek A et al	2025	Evaluating Microsoft Bing with ChatGPT-4 for the assessment of abdominal computed tomography and magnetic resonance images	Diagn Interv Radiol
71	Güneş YC et al	2025	Evaluating text and visual diagnostic capabilities of large language models on questions related to the Breast Imaging Reporting and Data System Atlas 5(th) edition	Diagn Interv Radiol
72	Kuzan BN et al	2025	A retrospective evaluation of the potential of ChatGPT in the accurate diagnosis of acute stroke	Diagn Interv Radiol
73	Koyun M et al	2025	Detection of Intracranial Hemorrhage from Computed Tomography Images: Diagnostic Role and Efficacy of ChatGPT-4o	Diagnostics
74	Ziegeler K et al	2025	Information Extraction from Lumbar Spine MRI Radiology Reports Using GPT4: Accuracy and Benchmarking Against Research-Grade Comprehensive Scoring	Diagnostics
75	Fabijan A et al	2023	Evaluating Scoliosis Severity Based on Posturographic X-ray Images Using a Contrastive Language-Image Pretraining Model	Diagnostics (Basel)
76	Fabijan A et al	2024	Artificial Intelligence in Medical Imaging: Analyzing the Performance of ChatGPT and Microsoft Bing in Scoliosis Detection and Cobb Angle Assessment	Diagnostics (Basel)
77	Lee KH et al	2024	ChatGPT's Accuracy on Magnetic Resonance Imaging Basics: Characteristics and Limitations Depending on the Question Type	Diagnostics (Basel)
78	Lee KH et al	2023	Validation of a Deep Learning Chest X-ray Interpretation Model: Integrating Large-Scale AI	Diagnostics (Basel)

			and Large Language Models for Comparative Analysis with ChatGPT	
79	Wada A et al	2024	Optimizing GPT-4 Turbo Diagnostic Accuracy in Neuroradiology through Prompt Engineering and Confidence Thresholds	Diagnostics (Basel)
80	Chung EM et al	2023	Feasibility and acceptability of ChatGPT generated radiology report summaries for cancer patients	Digit Health
81	Mohammadi M et al	2025	Diagnostic performance of ChatGPT in tibial plateau fracture in knee X-ray	Emerg Radiol
82	Chen Z et al	2025	Assessing the feasibility of ChatGPT-4o and Claude 3-Opus in thyroid nodule classification based on ultrasound images	Endocrine
83	Xia S et al	2025	Clinical application potential of large language model: a study based on thyroid nodules	Endocrine
84	Jiang H et al	2024	Transforming free-text radiology reports into structured reports using ChatGPT: A study on thyroid ultrasonography	Eur J Radiol
85	Busch F et al	2025	Multilingual feasibility of GPT-4o for automated Voice-to-Text CT and MRI report transcription	Eur J Radiol
86	Gupta M et al	2025	Large language models in radiology: Fluctuating performance and decreasing discordance over time	Eur J Radiol
87	Rau A et al	2024	Enhancing chatbot performance for imaging recommendations: Leveraging GPT-4 and context-awareness for trustworthy clinical guidance	Eur J Radiol
88	Hasani AM et al	2024	Evaluating the performance of Generative Pre-trained Transformer-4 (GPT-4) in standardizing radiology reports	Eur Radiol
89	Horiuchi D et al	2024	ChatGPT's diagnostic performance based on textual vs. visual information compared to radiologists' diagnostic performance in musculoskeletal radiology	Eur Radiol
90	Jeblick K et al	2024	ChatGPT makes medicine easy to swallow: an exploratory case study on simplified radiology reports	Eur Radiol
91	Rosen S et al	2024	Evaluating the reliability of ChatGPT as a tool for imaging test referral: a comparative study with a clinical decision support system	Eur Radiol
92	Siepmann R et al	2024	The virtual reference radiologist: comprehensive AI assistance for clinical image reading and interpretation	Eur Radiol
93	Brin D et al	2025	Assessing GPT-4 multimodal performance in radiological image analysis	Eur Radiol
94	Huppertz MS et al	2025	Revolution or risk?-Assessing the potential and challenges of GPT-4V in radiologic image interpretation	Eur Radiol
95	Langenbach MC et al	2025	Automated anonymization of radiology reports: comparison of publicly available natural language processing and large language models	Eur Radiol

96	Mitsuyama Y et al	2025	Comparative analysis of GPT-4-based ChatGPT's diagnostic performance with radiologists using real-world radiology reports of brain tumors	Eur Radiol
97	Woźnicki P et al	2025	Automatic structuring of radiology reports with on-premise open-source large language models	Eur Radiol
98	Maroncelli R et al	2024	Probing clarity: AI-generated simplified breast imaging reports for enhanced patient comprehension powered by ChatGPT-4o	Eur Radiol Exp
99	Butler JJ et al	2024	From jargon to clarity: Improving the readability of foot and ankle radiology reports with an artificial intelligence large language model	Foot Ankle Surg
100	Hong DR, Huang CY	2024	The performance of AI in medical examinations: an exploration of ChatGPT in ultrasound medical education	Front Med (Lausanne)
101	Huang J et al	2024	Feasibility of large language models for CEUS LI-RADS categorization of small liver nodules in patients at risk for hepatocellular carcinoma	Front Oncol
102	Fervers P et al	2024	ChatGPT yields low accuracy in determining LI-RADS scores based on free-text and structured radiology reports in German language	Front Radiol
103	Wang Y et al	2025	Evaluating the performance of ChatGPT in patient consultation and image-based preliminary diagnosis in thyroid eye disease	Frontiers in Medicine
104	Butler JJ et al	2024	Decoding Radiology Reports: Artificial Intelligence-Large Language Models Can Improve the Readability of Hand and Wrist Orthopedic Radiology Reports	Hand (N Y)
105	Hiredesai AN et al	2024	Is Artificial Intelligence the Future of Radiology? Accuracy of ChatGPT in Radiologic Diagnosis of Upper Extremity Bony Pathology	Hand (N Y)
106	Sarangi PK et al	2024	Radiological Differential Diagnoses Based on Cardiovascular and Thoracic Imaging Patterns: Perspectives of Four Large Language Models	Indian J Radiol Imaging
107	Sarangi PK et al	2024	Assessing the Capability of ChatGPT, Google Bard, and Microsoft Bing in Solving Radiology Case Vignettes	Indian J Radiol Imaging
108	Sarangi PK et al	2025	Evaluating ChatGPT-4's Performance in Identifying Radiological Anatomy in FRCR Part 1 Examination Questions	Indian J Radiol Imaging
109	Sarangi PK et al	2024	Radiologic Decision-Making for Imaging in Pulmonary Embolism: Accuracy and Reliability of Large Language Models-Bing, Claude, ChatGPT, and Perplexity	Indian J Radiol Imaging
110	Gupta A et al	2024	Enhancing Radiological Reporting in Head and Neck Cancer: Converting Free-Text CT Scan Reports to Structured Reports Using Large Language Models	Indian Journal of Radiology and Imaging
111	Gupta A et al	2024	Comparative Evaluation of Large Language Models for Translating Radiology Reports into Hindi	Indian Journal of Radiology and Imaging

112	Nishio M et al	2024	Fully automatic summarization of radiology reports using natural language processing with large language models	Informatics in Medicine Unlocked
113	Matute-González M et al	2024	Utilizing a domain-specific large language model for LI-RADS v2018 categorization of free-text MRI reports: a feasibility study	Insights Imaging
114	Sood A et al	2024	Generative pretrained transformer-4, an artificial intelligence text predictive model, has a high capability for passing novel written radiology exam questions	Int J Comput Assist Radiol Surg
115	Hu D et al	2024	Zero-shot information extraction from radiological reports using ChatGPT	Int J Med Inform
116	López-Úbeda P et al	2024	Evaluation of large language models performance against humans for summarizing MRI knee radiology reports: A feasibility study	Int J Med Inform
117	Zhu L et al	2024	Step into the era of large multimodal models: a pilot study on ChatGPT-4V(ision)'s ability to interpret radiological images	Int J Surg
118	Alibrahim Y et al	2025	AI speechbots and 3D segmentations in virtual reality improve radiology on-call training in resource-limited settings	Intelligence-Based Medicine
119	Lyu H et al	2024	Generative pretrained transformer 4: an innovative approach to facilitate value-based healthcare	Intelligent Medicine
120	Altalla' B et al	2025	Radiology Report Annotation Using Generative Large Language Models: Comparative Analysis	International Journal of Biomedical Imaging
121	Gordon EB et al	2024	Enhancing Patient Communication With Chat-GPT in Radiology: Evaluating the Efficacy and Readability of Answers to Common Imaging-Related Questions	J Am Coll Radiol
122	Kathait AS et al	2024	Assessing Laterality Errors in Radiology: Comparing Generative AI and Natural Language Processing	J Am Coll Radiol
123	Nazario-Johnson L et al	2023	Use of Large Language Models to Predict Neuroimaging	J Am Coll Radiol
124	Rao A et al	2023	Evaluating GPT as an Adjunct for Radiologic Decision Making: GPT-4 Versus GPT-3.5 in a Breast Imaging Pilot	J Am Coll Radiol
125	Zaki HA et al	2024	The Application of Large Language Models for Radiologic Decision Making	J Am Coll Radiol
126	Elhakim T et al	2025	Enhanced PROcedural Information READability for Patient-Centered Care in Interventional Radiology With Large Language Models (PRO-READ IR)	J Am Coll Radiol
127	Gordon EB et al	2025	Large Language Model Use in Radiology Residency Applications: Unwelcomed but Inevitable	J Am Coll Radiol
128	Gulati V et al	2024	Transcending Language Barriers: Can ChatGPT Be the Key to Enhancing Multilingual	J Am Coll Radiol

			Accessibility in Health Care?	
129	Kathait AS et al	2024	Assessing Laterality Errors in Radiology: Comparing Generative Artificial Intelligence and Natural Language Processing	J Am Coll Radiol
130	Woo KC et al	2024	Evaluation of GPT-4 ability to identify and generate patient instructions for actionable incidental radiology findings	J Am Med Inform Assoc
131	Yang F et al	2024	Large-Scale assessment of ChatGPT's performance in benign and malignant bone tumors imaging report diagnosis and its potential for clinical applications	J Bone Oncol
132	Williams MC et al	2023	How will artificial intelligence transform cardiovascular computed tomography? A conversation with an AI model	J Cardiovasc Comput Tomogr
133	Fabijan A et al	2024	Assessing the Accuracy of Artificial Intelligence Models in Scoliosis Classification and Suggested Therapeutic Approaches	J Clin Med
134	Lee RW et al	2024	Comparative Analysis of M4CXR, an LLM-Based Chest X-Ray Report Generation Model, and ChatGPT in Radiological Interpretation	J Clin Med
135	Wang WH et al	2024	An investigation study on the interpretation of ultrasonic medical reports using OpenAI's GPT-3.5-turbo model	J Clin Ultrasound
136	Ahyad RA et al	2024	Cutting Edge to Cutting Time: Can ChatGPT Improve the Radiologist's Reporting?	J Imaging Inform Med
137	Yasaka K et al	2024	Fine-Tuned Large Language Model for Extracting Patients on Pretreatment for Lung Cancer from a Picture Archiving and Communication System Based on Radiological Reports	J Imaging Inform Med
138	Kanemaru N et al	2025	The Fine-Tuned Large Language Model for Extracting the Progressive Bone Metastasis from Unstructured Radiology Reports	J Imaging Inform Med
139	Lyo S et al	2025	From Revisions to Insights: Converting Radiology Report Revisions into Actionable Educational Feedback Using Generative AI Models	J Imaging Inform Med
140	Miller L et al	2024	A Comparative Evaluation of Large Language Model Utility in Neuroimaging Clinical Decision Support	J Imaging Inform Med
141	Silbergleit M et al	2024	ChatGPT vs Gemini: Comparative Accuracy and Efficiency in CAD-RADS Score Assignment from Radiology Reports	J Imaging Inform Med
142	Su Y et al	2024	A Large Language Model to Detect Negated Expressions in Radiology Reports	J Imaging Inform Med
143	Yasaka K et al	2024	Classification of Interventional Radiology Reports into Technique Categories with a Fine-Tuned Large Language Model	J Imaging Inform Med
144	Hadi YH et al	2025	Enhancing CT examination efficiency with ChatGPT-4o for multilingual Hajj pilgrims: A short communication	J Med Imaging Radiat Sci

145	Busch F et al	2024	Integrating Text and Image Analysis: Exploring GPT-4V's Capabilities in Advanced Radiological Applications Across Subspecialties	J Med Internet Res
146	Chiu WHK et al	2024	Evaluating the Diagnostic Performance of Large Language Models on Complex Multimodal Medical Cases	J Med Internet Res
147	Ziegelmayer S et al	2023	Evaluation of GPT-4's Chest X-Ray Impression Generation: A Reader Study on Performance and Perception	J Med Internet Res
148	Zhang D et al	2024	Using Natural Language Processing (GPT-4) for Computed Tomography Image Analysis of Cerebral Hemorrhages in Radiology: Retrospective Analysis	J Med Internet Res
149	Chen TC et al	2024	Assessing the clinical reasoning of ChatGPT for mechanical thrombectomy in patients with stroke	J Neurointerv Surg
150	Fabijan A et al	2023	Artificial Intelligence in Scoliosis Classification: An Investigation of Language-Based Models	J Pers Med
151	Khanna P et al	2024	Artificial Intelligence in Multilingual Interpretation and Radiology Assessment for Clinical Language Evaluation (AI-MIRACLE)	J Pers Med
152	Han T et al	2024	Comparative Analysis of Multimodal Large Language Model Performance on Clinical Vignette Questions	Jama
153	Zhang Y et al	2025	AXpert: human expert facilitated privacy-preserving large language models for abdominal X-ray report labeling	JAMIA Open
154	Chen LC et al	2024	Assessing Large Language Models for Oncology Data Inference From Radiology Reports	JCO Clin Cancer Inform
155	Yamagishi Y et al	2025	Large Language Model Approach for Zero-Shot Information Extraction and Clustering of Japanese Radiology Reports: Algorithm Development and Validation	JMIR Cancer
156	Hirosawa T et al	2024	Evaluating ChatGPT-4's Diagnostic Accuracy: Impact of Visual Data Integration	JMIR Med Inform
157	Wu Q et al	2024	Evaluating Large Language Models for Automated Reporting and Data Systems Categorization: Cross-Sectional Study	JMIR Med Inform
158	Chang J et al	2024	Utilizing ChatGPT for Curriculum Learning in Developing a Clinical Grade Pneumothorax Detection Model: A Multisite Validation Study	Journal of Clinical Medicine
159	Koyun M, Taskent I	2025	Evaluation of Advanced Artificial Intelligence Algorithms' Diagnostic Efficacy in Acute Ischemic Stroke: A Comparative Analysis of ChatGPT-4o and Claude 3.5 Sonnet Models	Journal of Clinical Medicine
160	Öztürk A et al	2025	Can Gpt-4o Accurately Diagnose Trauma X-Rays? A Comparative Study with Expert Evaluations	Journal of Emergency Medicine
161	Dehdab R et al	2024	Evaluating ChatGPT-4V in chest CT diagnostics: a critical image interpretation assessment	Jpn J Radiol
162	Hirano Y et al	2024	GPT-4 Turbo with Vision fails to outperform text-only GPT-4 Turbo in the Japan Diagnostic	Jpn J Radiol

			Radiology Board Examination	
16 3	Nakaura T et al	2024	Preliminary assessment of automated radiology report generation with generative pre-trained transformers: comparing results to radiologist-generated reports	Jpn J Radiol
16 4	Oura T et al	2024	Diagnostic accuracy of vision-language models on Japanese diagnostic radiology, nuclear medicine, and interventional radiology specialty board examinations	Jpn J Radiol
16 5	Sonoda Y et al	2024	Diagnostic performances of GPT-4o, Claude 3 Opus, and Gemini 1.5 Pro in "Diagnosis Please" cases	Jpn J Radiol
16 6	Toyama Y et al	2024	Performance evaluation of ChatGPT, GPT-4, and Bard on the official board examination of the Japan Radiology Society	Jpn J Radiol
16 7	Harigai A et al	2025	Response accuracy of GPT-4 across languages: insights from an expert-level diagnostic radiology examination in Japan	Jpn J Radiol
16 8	Kurokawa R et al	2024	Diagnostic performances of Claude 3 Opus and Claude 3.5 Sonnet from patient history and key images in Radiology's "Diagnosis Please" cases	Jpn J Radiol
16 9	López-Úbeda P et al	2025	The added value of including thyroid nodule features into large language models for automatic ACR TI-RADS classification based on ultrasound reports	Jpn J Radiol
17 0	Sonoda Y et al	2025	Structured clinical reasoning prompt enhances LLM's diagnostic capabilities in diagnosis please quiz cases	Jpn J Radiol
17 1	Suzuki K et al	2025	Preliminary assessment of TNM classification performance for pancreatic cancer in Japanese radiology reports using GPT-4	Jpn J Radiol
17 2	Tozuka R et al	2025	Application of NotebookLM, a large language model with retrieval-augmented generation, for lung cancer staging	Jpn J Radiol
17 3	Lim B et al	2024	Evaluating Artificial Intelligence's Role in Teaching the Reporting and Interpretation of Computed Tomographic Angiography for Preoperative Planning of the Deep Inferior Epigastric Artery Perforator Flap	JPRAS Open
17 4	Butler JJ et al	2024	From technical to understandable: Artificial Intelligence Large Language Models improve the readability of knee radiology reports	Knee Surg Sports Traumatol Arthrosc
17 5	Kim H et al	2024	ChatGPT Vision for Radiological Interpretation: An Investigation Using Medical School Radiology Examinations	Korean J Radiol
17 6	Gu K et al	2024	Using GPT-4 for LI-RADS feature extraction and categorization with multilingual free-text reports	Liver Int
17 7	Sheng L et al	2025	Large Language Models for Diagnosing Focal Liver Lesions From CT/MRI Reports: A Comparative Study With Radiologists	Liver International
17	Bigolin Lanfredi R	2025	Enhancing chest X-ray datasets with privacy-	Med Image Anal

8	et al		preserving large language models and multi-type annotations: A data-driven approach for improved classification	
179	Erez E et al	2024	Thoracic Aorta Measurement Extraction from Computed Tomography Radiology Reports Using Instruction Tuned Large Language Models	medRxiv
180	Foltyn-Dumitru M et al	2025	The potential of GPT-4 advanced data analysis for radiomics-based machine learning models	Neurooncol Adv
181	D'Anna G et al	2024	Can large language models pass official high-grade exams of the European Society of Neuroradiology courses? A direct comparison between OpenAI chatGPT 3.5, OpenAI GPT4 and Google Bard	Neuroradiology
182	Horiuchi D et al	2024	Accuracy of ChatGPT generated diagnosis from patient's medical history and imaging findings in neuroradiology cases	Neuroradiology
183	Kanzawa J et al	2024	Automated classification of brain MRI reports using fine-tuned large language models	Neuroradiology
184	Das A et al	2025	Weakly supervised language models for automated extraction of critical findings from radiology reports	npj Digital Medicine
185	Kim SH et al	2025	Benchmarking the diagnostic performance of open source LLMs in 1933 Eurorad case reports	npj Digital Medicine
186	Mohammad-Rahimi H et al	2024	Performance of AI chatbots on controversial topics in oral medicine, pathology, and radiology	Oral Surg Oral Med Oral Pathol Oral Radiol
187	Tepe M et al	2024	Decoding medical jargon: The use of AI language models (ChatGPT-4, BARD, microsoft copilot) in radiology reports	Patient Educ Couns
188	Reith TP et al	2024	Capability of multimodal large language models to interpret pediatric radiological images	Pediatr Radiol
189	Park HJ et al	2024	Extraction of clinical data on major pulmonary diseases from unstructured radiologic reports using a large language model	PLoS One
190	Kufel J et al	2023	Will ChatGPT pass the Polish specialty exam in radiology and diagnostic imaging? Insights into strengths and limitations	Pol J Radiol
191	Wang Z et al	2024	Assessing the role of GPT-4 in thyroid ultrasound diagnosis and treatment recommendations: enhancing interpretability with a chain of thought approach	Quant Imaging Med Surg
192	Sorin V et al	2024	Generative pre-trained transformer (GPT)-4 support for differential diagnosis in neuroradiology	Quant Imaging Med Surg
193	Sun P et al	2024	Preliminary experiments on interpretable ChatGPT-assisted diagnosis for breast ultrasound radiologists	Quant Imaging Med Surg
194	Choi HS et al	2023	Developing prompts from large language model for extracting clinical information from pathology and ultrasound reports in breast cancer	Radiat Oncol J
19	Almeida LC et al	2024	Performance of ChatGPT on the Brazilian	Radiol Artif Intell

5			Radiology and Diagnostic Imaging and Mammography Board Examinations	
19 6	Le Guellec B et al	2024	Performance of an Open-Source Large Language Model in Extracting Information from Free-Text Radiology Reports	Radiol Artif Intell
19 7	Schmidt RA et al	2024	Generative Large Language Models for Detection of Speech Recognition Errors in Radiology Reports	Radiol Artif Intell
19 8	Leitão CA et al	2024	Performance of ChatGPT on questions from the Brazilian College of Radiology annual resident evaluation test	Radiol Bras
19 9	Mallio CA et al	2023	Large language models for structured reporting in radiology: performance of GPT-4, ChatGPT-3.5, Perplexity and Bing	Radiol Med
20 0	Irmici G et al	2024	How do large language models answer breast cancer quiz questions? A comparative study of GPT-3.5, GPT-4 and Google Gemini	Radiol Med
20 1	Salvador R et al	2024	Comparing ChatGPT and medical student performance in a real image-based Radiology and Applied Physics in Medicine exam	Radiologia
20 2	Adams LC et al	2023	Leveraging GPT-4 for Post Hoc Transformation of Free-text Radiology Reports into Structured Reporting: A Multilingual Feasibility Study	Radiology
20 3	Amin KS et al	2023	Accuracy of ChatGPT, Google Bard, and Microsoft Bing for Simplifying Radiology Reports	Radiology
20 4	Bhayana R et al	2023	GPT-4 in Radiology: Improvements in Advanced Reasoning	Radiology
20 5	Bhayana R et al	2023	Performance of ChatGPT on a Radiology Board-style Examination: Insights into Current Strengths and Limitations	Radiology
20 6	Bhayana R et al	2024	Large Language Models for Automated Synoptic Reports and Resectability Categorization in Pancreatic Cancer	Radiology
20 7	Cozzi A et al	2024	BI-RADS Category Assignments by GPT-3.5, GPT-4, and Google Bard: A Multilanguage Study	Radiology
20 8	Doshi R et al	2024	Quantitative Evaluation of Large Language Models to Streamline Radiology Report Impressions: A Multimodal Retrospective Analysis	Radiology
20 9	Fink MA et al	2023	Potential of ChatGPT and GPT-4 for Data Mining of Free-Text CT Reports on Lung Cancer	Radiology
21 0	Gertz RJ et al	2023	GPT-4 for Automated Determination of Radiological Study and Protocol based on Radiology Request Forms: A Feasibility Study	Radiology
21 1	Gertz RJ et al	2024	Potential of GPT-4 for Detecting Errors in Radiology Reports: Implications for Reporting Accuracy	Radiology
21 2	Haver HL et al	2023	Appropriateness of Breast Cancer Prevention and Screening Recommendations Provided by ChatGPT	Radiology
21	Jankowski S et al	2024	ChatGPT versus Radiology Institutional	Radiology

3			Websites: Comparative Analysis of Radiation Protection Information Provided to Patients	
214	Kottlors J et al	2023	Feasibility of Differential Diagnosis Based on Imaging Patterns Using a Large Language Model	Radiology
215	Krishna S et al	2024	Evaluation of Reliability, Repeatability, Robustness, and Confidence of GPT-3.5 and GPT-4 on a Radiology Board-style Examination	Radiology
216	Laukamp KR et al	2024	Monitoring Patients with Glioblastoma by Using a Large Language Model: Accurate Summarization of Radiology Reports with GPT-4	Radiology
217	Lehnen NC et al	2024	Data Extraction from Free-Text Reports on Mechanical Thrombectomy in Acute Ischemic Stroke Using ChatGPT: A Retrospective Analysis	Radiology
218	Li D et al	2024	Comparing GPT-3.5 and GPT-4 Accuracy and Drift in Radiology Diagnosis Please Cases	Radiology
219	Mukherjee P et al	2023	Feasibility of Using the Privacy-preserving Large Language Model Vicuna for Labeling Radiology Reports	Radiology
220	Rahsepar AA et al	2023	How AI Responds to Common Lung Cancer Questions: ChatGPT vs Google Bard	Radiology
221	Rau A et al	2023	A Context-based Chatbot Surpasses Trained Radiologists and Generic ChatGPT in Following the ACR Appropriateness Guidelines	Radiology
222	Suh PS et al	2024	Comparing Diagnostic Accuracy of Radiologists versus GPT-4V and Gemini Pro Vision Using Image Inputs from Diagnosis Please Cases	Radiology
223	Ueda D et al	2023	ChatGPT's Diagnostic Performance from Patient History and Imaging Findings on the Diagnosis Please Quizzes	Radiology
224	Wu SH et al	2024	Collaborative Enhancement of Consistency and Accuracy in US Diagnosis of Thyroid Nodules Using Large Language Models	Radiology
225	Zhou Y et al	2024	Evaluating GPT-V4 (GPT-4 with Vision) on Detection of Radiologic Findings on Chest Radiographs	Radiology
226	Bhayana R et al	2024	Retrieval-Augmented Generation for Large Language Models in Radiology: Another Leap Forward in Board Examination Performance	Radiology
227	Doo FX et al	2024	Optimal Large Language Model Characteristics to Balance Accuracy and Energy Use for Sustainable Medical Applications	Radiology
228	Dorfner FJ et al	2024	Comparing Commercial and Open-Source Large Language Models for Labeling Chest Radiograph Reports	Radiology
229	Hayden N et al	2024	Performance of GPT-4 with Vision on Text- and Image-based ACR Diagnostic Radiology In-Training Examination Questions	Radiology
230	Meddeb A et al	2024	Large Language Model Ability to Translate CT and MRI Free-Text Radiology Reports Into Multiple Languages	Radiology

23<br>1	Mukherjee P et al	2024	Evaluation of GPT Large Language Model Performance on RSNA 2023 Case of the Day Questions	Radiology
23<br>2	Suh PS et al	2024	Comparing Large Language Model and Human Reader Accuracy with New England Journal of Medicine Image Challenge Case Image Inputs	Radiology
23<br>3	Sun SH et al	2024	Testing the Ability and Limitations of ChatGPT to Generate Differential Diagnoses from Transcribed Radiologic Findings	Radiology
23<br>4	Zhang L et al	2024	Constructing a Large Language Model to Generate Impressions from Findings in Radiology Reports	Radiology
23<br>5	Gotta J et al	2024	Large language models (LLMs) in radiology exams for medical students: Performance and consequences	Rofo
23<br>6	Park J et al	2024	Patient-centered radiology reports with generative artificial intelligence: adding value to radiology reporting	Sci Rep
23<br>7	Russe MF et al	2023	Performance of ChatGPT, human radiologists, and context-aware ChatGPT in identifying AO codes from radiology reports	Sci Rep
23<br>8	Ajmera P et al	2024	Validity of ChatGPT-generated musculoskeletal images	Skeletal Radiol
23<br>9	Kuckelman IJ et al	2024	Translating musculoskeletal radiology reports into patient-friendly summaries using ChatGPT-4	Skeletal Radiol
24<br>0	Hallinan J et al	2025	MRI spine request form enhancement and auto protocoling using a secure institutional large language model	Spine J
24<br>1	Jenko N et al	2024	An evaluation of AI generated literature reviews in musculoskeletal radiology	Surgeon
24<br>2	Liu C et al	2024	Harnessing Large Language Models for Structured Reporting in Breast Ultrasound: A Comparative Study of Open AI (GPT-4.0) and Microsoft Bing (GPT-4)	Ultrasound Med Biol
24<br>3	Cole MW et al	2025	Accuracy of a ChatGPT in Diagnosing Urologic Conditions From Cross-sectional Imaging	Urology
24<br>4	Chen Z et al	2024	IQAGPT: computed tomography image quality assessment with vision-language and ChatGPT models	Vis Comput Ind Biomed Art
24<br>5	Yuan LH et al	2024	The In-depth Comparative Analysis of Four Large Language AI Models for Risk Assessment and Information Retrieval from Multi-Modality Prostate Cancer Work-up Reports	World J Mens Health
24<br>6	Almekkawi AK et al	2025	Comparative Analysis of Large Language Models and Spine Surgeons in Surgical Decision-Making and Radiological Assessment for Spine Pathologies	World Neurosurg

Supplementary Table S2. Comparison of adherence to key elements required for LLM research according to study design.

Section and Topic	Details	No. of reported articles (percentage)			*P* value*
		Total (n=246)	Retro (n=87)	Pro (n=15)	
Study Methodology					
Study design	Did the study specify whether it was retrospective or prospective?	102 (41.5)			
Retrospective		87 (85.3)			
Prospective		15 (14.7)			
IRB	Was IRB approval obtained or waived?	177 (72.0)	78 (89.7)	10 (66.7)	0.02
Obtained		92 (52.0)	56 (71.8)	4 (40.0)	0.04
Waived		85 (48.0)	22 (28.2)	6 (60.0)	0.04
Test dataset					
Public	Publicly available open-source data	99 (40.2)	21 (24.1)	9 (60.0)	0.005
Hospital	Real-world hospital data	102 (41.5)	60 (69.0)	4 (26.7)	**0.002**
Generated	Generated data	44 (17.9)	7 (8.0)	4 (26.7)	0.03
Others	Using publicly unavailable data or generating outputs without test dataset	10 (4.1)	0 (0)	1 (6.7)	0.35

Large Language Models					
Model name	Did the study specify the name of the model used?	246 (100)	87 (100)	15 (100)	>0.99
Model version	Did the study specify the exact version of the model used?	68 (27.6)	30 (34.5)	4 (26.7)	0.56
Accessing date	Did the study mention the date when the LLM was run?	88 (35.8)	26 (29.9)	8 (53.3)	0.08
API	Did the study mention whether an API was used?	62 (25.2)	28 (32.2)	5 (33.3)	0.93
Prompts					
Full prompts	Did the study provide the full prompts used in the research?	101 (41.1)	41 (47.1)	5 (33.3)	0.32
Partially provided	Providing examples of prompts without including the full prompts.	81 (32.9)	26 (29.9)	4 (26.7)	0.80
Not provided		64 (26.0)	20 (23.0)	6 (40.0)	0.17
Output probability					
Repetition	Did the study specify the number of querying attempts?	56 (22.8)	21 (24.1)	5 (33.3)	0.45
Repeated		51 (91.1)	21 (100)	5 (100)	>0.99
Multiple results analysis	If repeated, did the study explain how the multiple results were synthesized for analysis?	42 (82.4)	16 (76.2)	3 (60.0)	0.05
Reliability analysis	If repeated, did the study analyze the reliability between responses?	28 (54.9)	14 (66.7)	2 (40.0)	0.28
Temperature	Was there mention of the temperature setting used?	34 (13.8)	15 (17.2)	1 (6.7)	0.30
Comparison					
Human readers	Did the study compare the results with human readers?	47 (19.1)	26 (29.9)	2 (13.3)	0.19
Blinding of readers	If human readers were involved, did the study mention whether	20 (42.6)	12 (46.2)	0 (0)	0.21

	they were blinded to the data and how blinding was done?				
Multiple comparison	Were three or more readers (LLM + human) involved in the comparison?	82 (33.3)	36 (41.4)	3 (20.0)	0.12
Statistical method	If multiple readers were involved, did the study consider a statistical method for multiple comparison?	11 (13.4)	4 (11.1)	1 (33.3)	0.28
p-value correction	If multiple readers were involved, did the study adjusted p-value?	15 (18.3)	9 (25.0)	0 (0)	0.33

[*] *p* -value for comparison between retrospective and prospective studies using chi-square test. A *p*-value less than 0.002 (0.05/24) was considered statistically significant after Bonferroni correction. API = application programming interface, IRB = institutional review board, LLM = large language model

Supplementary Analysis of Inter-Rater Reliability

To evaluate the consistency of data extraction among reviewers, we conducted a supplementary inter-rater reliability analysis using a representative subset of 29 articles published in *Radiology*. Each article was re-assessed independently by a reviewer who had not participated in the initial data extraction of the specific section. The frequency of initial agreement across reviewers was quantified as the agreement incidence, which averaged approximately 97% overall, demonstrating very high consistency in assessments. Agreement incidence for specific items was as follows: evaluation (Table 3), 96.6%; test dataset (Table 1), 96.6%; prompt (Table 1), 93.1%; and all other items—including study category, methodology, large language models except prompt, output probability, and comparison—100%. These findings indicate strong reproducibility of data extraction decisions. Additionally, all reviewers underwent initial calibration sessions before the original data extraction to harmonize coding criteria and ensure methodological consistency.
